# Flame-Retardant Wood Scrimber/Plywood Composites: Preparation, Characterization, and Enhanced Structural Performance

**DOI:** 10.3390/polym17182556

**Published:** 2025-09-22

**Authors:** Liyuan Yao, Feifan Song, Ming Wei, Aijuan Wang, Xiaonan Xu, Zhilin Chen, Rui Rong, Peng Jiang

**Affiliations:** 1 School of Emergency Management and Safety Engineering, China University of Mining and Technology, Beijing 100083, China; rebecca_yly@163.com (L.Y.); r3361510871@163.com (R.R.); 2 Research Institute of Wood Industry, Chinese Academy of Forestry, Xiangshan Road, Haidian District, Beijing 100091, China; 3 Shandong Xingang Enterprise Group Co., Ltd., Nanfang Road, Lanshan District, Linyi 276002, China; 4 INSA Centre Val de Loire, University Orléans, PRISME, EA 4229, F-18022 Bourges, France

**Keywords:** veneer-based wood composites, wood scrimber, plywood, flame-retardant mechanism

## Abstract

Veneer-based wood composites are widely used for interior applications, yet their high flammability and smoke emission significantly limit their safe use in buildings. In this study, a multifunctional flame-retardant polyethylene adhesive film was developed via melt blending and hot pressing of a mixture of amino trimethylene phosphonic acid (ATMP), hydroxyethylidene diphosphonic acid (HDEP), melamine (MEL), and sodium alginate (SA). This film was laminated onto veneers to fabricate flame-retardant decorative plywood. Simultaneously, wood scrimber units for structural applications were prepared by impregnating wood with a flame-retardant system consisting of sodium silicate (Ss) and sodium tetraborate (St). These treated components were integrated to form a flame-retardant wood scrimber/plywood composite (AHM-S), with the wood scrimber as the core layer and the treated plywood as surface layers. Compared to the control, the AHM-S composite showed a 44.1% reduction in the second peak heat release rate (pk-HRR2), a 22.6% decrease in total heat release (THR), and a 12.7% reduction in maximum flame spread distance (MD_300°C_). Moreover, the time to reach 275 °C on the unexposed side (T_275°C_) was extended by 90.2%. These improvements are attributed to the synergistic flame-retardant effects of the surface film and impregnated core, which jointly suppress flame spread and delay thermal degradation. The composite demonstrates promising fire safety and mechanical performance for engineered wood applications.

## 1. Introduction

Wood, as a natural and renewable polymeric material, possesses significant low-carbon advantages compared with high-energy-consumption construction materials such as steel and cement [1,2]. Its processing, transportation, and installation require considerably less energy, thereby substantially reducing the carbon footprint over the entire life cycle. In applications such as construction and furniture, wood not only continuously sequesters carbon during its service life but also, after disposal, can be recycled or converted into bioenergy to delay or even prevent carbon release, effectively substituting for high-energy, high-emission traditional materials and demonstrating outstanding low-carbon utilization value. However, high-quality large-diameter timber resources are relatively limited, and natural wood often falls short of meeting modern demands for dimensional stability, mechanical performance, and multifunctionality. To better exploit the potential of wood, it has been processed into veneers, wood particles, fibers, and other wood-based units for the manufacture of various engineered wood products, thereby substantially improving wood utilization efficiency while imparting the resulting materials with characteristics typical of composite materials [3,4]. Depending on the type of wood unit employed, engineered wood products are generally classified into particleboard, fiberboard, and veneer-based boards, among which veneer-based boards currently account for the largest share of consumption. Plywood and wood scrimber, as representative veneer-based products, are fabricated using different processing techniques and exhibit distinct mechanical properties; this diversity, however, enables complementary advantages within composite systems, better fulfilling the requirements of multifunctional integration characteristic of modern composite materials [5].

Plywood and wood scrimber, as typical veneer-based wood composites, are primarily composed of wood and have been widely applied in the construction and furniture sectors [6]. Plywood features a smooth surface and relatively high strength, with perpendicular arrangement of veneer grains across layers, providing balanced mechanical properties and dimensional stability, making it suitable for furniture and interior decoration. In comparison, wood scrimber is fabricated by recombining fibers or veneers into an optimized structure, offering enhanced stability and higher strength, and is suitable for load-bearing components and large-scale furniture [7]. Under the trends of green building and sustainable design, the complementary performance achieved through composite fabrication of plywood and wood scrimber has garnered considerable attention, as these composites exhibit light weight, high strength, and environmentally friendly characteristics. Nevertheless, wood-based components are inherently flammable, and their combustion can release toxic gases, posing significant fire safety hazards [8,9]. With increasingly stringent global fire safety regulations for building materials—including Europe’s EN 13501-1 [10], China’s GB 8624 [11], and relevant standards in the United States and Japan—flame-retardant modification has become a critical pathway to enhance the safety performance of wood composites, making the development of such materials of substantial economic and social significance.

Enhancing the fire resistance of plywood is primarily achieved through impregnation, surface coating [12,13,14,15], or the use of flame-retardant adhesives. The impregnation method enables flame retardants to penetrate wood pores but often suffers from hygroscopicity and degradation [16]. Surface coating can impair appearance and loses effectiveness once the outer layer is damaged. Consequently, recent research has focused on incorporating flame retardants into adhesive systems, which preserves esthetics while improving overall flame resistance. For example, BSPC-ADH adhesives demonstrated both excellent flame retardancy and water resistance [17]. Gao et al. developed a phytic acid/b-PEI adhesive, yielding wood products with an exceptionally high LOI (100%) and significantly suppressed heat and smoke release [18]. Wu et al. reported a phosphorus–nitrogen adhesive combined with melamine–urea–formaldehyde (MUF) resin, reducing total heat release by 71% compared with neat plywood [19]. Thus, the development of flame-retardant adhesives represents an effective strategy for manufacturing high-performance fire-resistant plywood. Currently, a wide range of flame-retardant systems have been introduced into adhesives, including inorganic minerals (e.g., kaolinite, wollastonite, bentonite) [20,21,22], layered double hydroxides (LDH) such as Zn(OH)_2_ and Al(OH)_3_ [23], silicon-based compounds [24]. Among these, phosphorus–nitrogen flame retardants (e.g., ATMP, HEDP) are particularly attractive due to their halogen-free nature, high efficiency, and dual-phase (gas–condensed) flame-retardant mechanism. By promoting the formation of a porous char layer, they provide thermal insulation, oxygen shielding, and smoke suppression, while remaining cost-effective and scalable [25].

Since the fabrication of engineered wood involves recombination of fibers or veneers with adhesives, the adhesive properties critically determine final product quality. Flame retardants used in adhesives should therefore minimize adverse effects on mechanical strength and structural integrity. Traditional impregnation treatments with melamine, inorganic phosphates, boric acid, or ammonium dihydrogen phosphate can enhance flame resistance but are prone to leaching and environmental degradation [26,27,28,29,30]. Silicon-based compounds (e.g., siloxanes, silica) are widely employed to improve dimensional stability and mechanical strength [31,32,33]; however, their tendency to remain on the surface during combustion limits their penetration and flame-retardant effectiveness [34,35]. To address this, silicon compounds are often combined with other flame retardants [34,35]. For instance, nano-silica blended with ammonium polyphosphate (2:3 ratio) increased the LOI of LFI-PE composites from 19.6% to 23.3% [36]; organic silicone resin treatment raised LOI from 14.6% to 26–29% while reducing the peak heat release rate (pk-HRR) [37]; and lignosulfonate–aluminum phosphate coatings achieved an LOI of 27.2% [38]. Sodium silicate nonahydrate, a widely available, low-cost soluble silicate, has been applied across multiple fields [39], while sodium tetraborate decahydrate, an important boron-containing compound, offers low toxicity, high solubility, stability, and long-term modification benefits without releasing corrosive gases [40]. Recent studies highlight the potential of such compounds: Sun [41] developed Al–Si–EPW composites using silicate, aluminum sulfate, and waterborne epoxy, which reduced pk-HRR and peak smoke release rates by 39.04% and 34.68%, respectively, and delayed their occurrence by 100 s. Nguyen [42] reported that sodium silicate combined with styrene–acrylic copolymer (SAC) improved flame resistance, ignition delay, LOI, and emission reduction compared with SAC alone. However, the synergistic effects of sodium silicate and sodium tetraborate in engineered wood remain insufficiently explored, representing a promising avenue for future research.

At present, studies on flame-retardant materials prepared by combining the two approaches remain limited. Sun Lichao [43] studied wood fiber–polypropylene composites (WPPC) and proposed a layered strategy using expandable graphite on the surface and ammonium polyphosphate on the bottom layer, which markedly improved flame retardancy and smoke suppression. Tian F [44] constructed a three-layer structure (“upper surface–core–lower surface”) for large particleboards and applied TBC at different mass ratios to each layer, achieving notable improvements in internal bond strength (up to +0.52 MPa), static bending strength (+18.35–30.01 MPa), moisture resistance, and flame retardancy. Lu J [45], based on the composition and structural characteristics of poplar plywood, proposed a layered flame-retardant method in which the surface layer suppresses flame spread and the subsurface layer hinders flame penetration. During combustion, a uniform and dense phospho-carbonaceous foamed char layer was formed, effectively insulating heat and oxygen, thereby inhibiting the decomposition and combustion of the underlying material. However, few studies have considered the incorporation of flame retardants into load-bearing structural members while simultaneously imparting decorative functionality. Given the high plasticity and unique architectural expressiveness of wood, some researchers have suggested using timber structural elements to achieve both mechanical load-bearing and architectural esthetics. Research in this area has been strengthened both domestically and internationally, leading to the successful development of decorative wooden wall panels that combine veneers with solid wood, which serve load-bearing purposes while reducing production costs and providing decorative features [46], Nevertheless, research on integrating wood scrimber with plywood to achieve this dual functionality remains scarce.

Here, we design a decorative layered flame-retardant structural element. Wood scrimber served as the core to ensure strength and was impregnated with a flame-retardant solution. Veneer layers were attached to both sides, with the outer surface laminated with a flame-retardant polyethylene film possessing an intumescent flame-retardant mechanism, thereby constructing an integrated layered flame-retardant system [47]. This design not only combines the advantages of wood scrimber and plywood to reduce production costs but also facilitates independent modification and functionalization of both the core and surface layers, thereby enhancing overall performance and meeting diverse application requirements. Flame-retardant films were prepared by compounding aminotris (methylenephosphonic acid) (ATMP), hydroxyethylidene diphosphonic acid (HEDP), and melamine (MEL) with sodium alginate, while the core was treated with sodium metasilicate nonahydrate and sodium tetraborate. Furthermore, the material was systematically compared with control samples treated with conventional flame retardants (e.g., aluminum hydroxide, ammonium sulfate) in terms of flame-retardant and smoke-suppression performance, mechanical strength, and high-temperature degradation behavior. Flame spread and penetration behavior were characterized using a small-scale temperature distribution measurement platform, complemented by high-temperature charring observation, mechanical testing, SEM-EDS microstructural analysis, FTIR and XPS, and comprehensive evaluation of flame-retardant and smoke-suppression performance. Results show that this layered strategy significantly enhances fire resistance and smoke suppression without compromising mechanical strength, indicating strong potential for building and interior applications.

## 2. Experiments and Methods

### 2.1. Materials

Amino trimethylene phosphonic acid (ATMP) and hydroxyethylidene diphosphonic acid (HEDP) were supplied by Shandong Taihe Technology Co., Ltd. (No. 1, Shiliquan East Road, Shizhong District, Zaozhuang City, Shandong Province, China). High-density polyethylene (HF5110) and low-density polyethylene (LD402) were purchased from the National Petrochemical Company of Iran (Tehran Province, Tehran, No.144, North, Sheykh Bahayi St, Iran). Commercial thermoplastic adhesive film (thickness: 0.1 mm) was obtained from Fujian Qingxin Technology Co., Ltd. (No. 6, Dongshi East Avenue, Tanshi Town, Minhou County, Fuzhou City, Fujian Province, China), while decorative veneers (thickness: 0.1 mm) were supplied by Hanshifu Integrated Home Furnishing Co., Ltd. (Economic Development Zone, Lanshan District, Linyi City, Shandong Province, China). Sodium alginate (SA, analytical grade ≥ 99%) was purchased from Merck (Darmstadt, Germany). Melamine (MEL), pentaerythritol (PER), sodium silicate (Ss, analytical grade ≥ 99%), sodium tetraborate (St, analytical grade ≥ 99%), aluminum hydroxide (Al(OH)_3_, analytical grade ≥ 99%), and ammonium sulfate ((NH_4_)_2_SO_4_, analytical grade ≥99%) were obtained from Shanghai Macklin Biochemical Co., Ltd. (Building 1, No. 68 Huatuo Road, Zhangjiang Hi-Tech Park, Pudong New District, Shanghai, China). Eucalyptus wood (Eucalyptus grandis × urophylla, 8 years old, density ≈ 0.6 g/cm^3^) was sourced from Liuzhou, Guangxi, China. Wood scrimber units were prepared by fiber disintegration and supplied by Suzhou Suzhi Environmental Protection Technology Co., Ltd. (Room 13, Building 26, Federal International Business Garden, Yushan Town, Kunshan City, Suzhou City, Jiangsu Province, China). with an absolute dry moisture content of 10%. Phenolic resin was provided by Aecter New Materials (Guangdong) Co., Ltd. (No. 222, West Jinyuan Avenue, Xuzhen Community, Jinli Town, Gaoyao District, Zhaoqing City, Guangdong Province, China) (solid content: 52.4%, viscosity: 73 cps/25 °C, pH = 9.84, free phenol content = 1.89%).

### 2.2. Sample and Composite Preparation

Synthesis of the AHM flame retardant: ATMP-HEDP (2:1, 16 g) was added into 100 g of an 8 wt% melamine aqueous solution under stirring at 100 °C. The mixture was cooled and filtered under vacuum to obtain solid products with a yield of 80%. The collected solids were dried at 80 °C for 4 h in an oven, resulting in white crystalline AHM flame-retardant powder.

**Figure 1:**
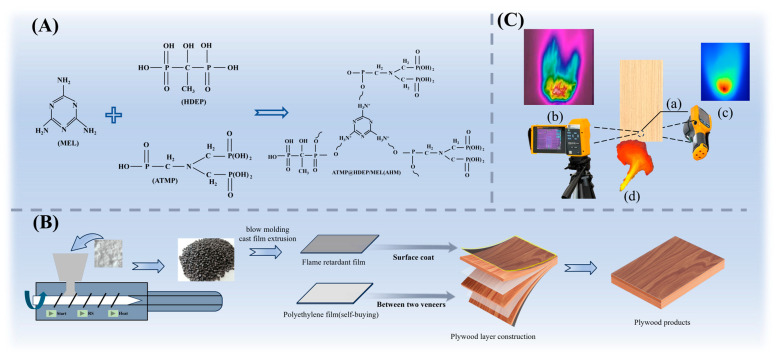
AHM Synthesis Scheme (**A**); Preparation process (**B**); Physical drawing of fire spreading test platform (**C**), Ignition Point (**a**), Front-face Temperature Variation and Fire Spread (**b**), Back-face Temperature Variation and Infrared Thermal Imaging (**c**), and Constant Heat Source (Propane Gas) (**d**).

Preparation of flame-retardant thermoplastic films (AHM-S): AHM was used as the main acid source and combined with MEL and SA at a mass ratio of 3:1:1 to form the AHM-S flame-retardant system. The flame retardant (25 wt%) was incorporated into a polyethylene blend consisting of LDPE (LD402) and HDPE (HF5110) at a 7:3 mass ratio. The mixture was melt-blended and pelletized, followed by extrusion at 180 °C and 5 r/min using a single-screw extruder. The extrudates were crushed and hot-pressed at 130 °C and 5 MPa for 10 min to form a 0.2 mm thick flame-retardant polyethylene film, as illustrated in Figure 1A,B.

Preparation of Ss–St/ammonium sulfate impregnation solution: A 10 wt% aqueous flame-retardant solution was prepared by dissolving 200 g of 5 wt% sodium silicate (Ss) in deionized water at 60 °C under stirring, followed by the gradual addition of 200 g of 5 wt% sodium tetraborate (St). A 10 wt% aqueous solution was obtained by dissolving 200 g of 5 wt% ammonium sulfate ((NH_4_)_2_SO_4_) in deionized water at 60 °C under stirring, followed by concentration adjustment with water.

Preparation of flame-retardant wood scrimber core: Eucalyptus recombinant units were immersed in 10 wt% Ss–St or 10 wt% (NH_4_)_2_SO_4_ solutions at room temperature for 3 h, drained, and oven-dried to a moisture content of 8–10%. The units were subsequently impregnated with 15 wt% phenolic resin solution, dried again, and then aligned longitudinally in a mold. The composites were hot-pressed at 150 °C and 10 MPa for 10 min to form wood scrimber boards (300 mm × 160 mm × 5 mm).

Preparation of wood scrimber/plywood composites (AHM-S type): Flame-retardant polyethylene films (AHM-S) were placed on the outer surfaces of a three-ply cross-laminated veneer assembly. Decorative veneers were positioned between the films and the wood scrimber core. The assemblies were hot-pressed at 140 °C and 8 MPa for 8 min (1 min/mm thickness), followed by cold pressing at 5 MPa for 4 min, yielding composite panels (300 mm × 160 mm × 8 mm).

Preparation of control composites: For Al(OH)_3_-based flame-retardant plywood, the AHM-S films were replaced with Al(OH)_3_–PE films (Preparation of Al(OH)_3_-based flame-retardant films: Al(OH)_3_ (25 wt%) was incorporated into the same polyethylene base formulation (LDPE:HDPE = 7:3) and hot-pressed into 0.2 mm thick flame-retardant films). For neat plywood, the outer flame-retardant films were replaced with neat polyethylene films, while the wood scrimber core was maintained. All samples were fabricated under identical hot-pressing and cold-pressing conditions.

The formulations for layered flame-retardant treatments have been listed in Table 1.

### 2.3. Characterization

Flame spread and penetration test: The flame spread tests were conducted on a custom-built small-scale combustion platform, as illustrated in Figure 1C. During testing, the long side of the composite sample was oriented vertically, with a horizontal reference line located 3 cm above the bottom edge and a vertical axis passing through the midpoint of the short edge. The intersection of these two axes was defined as the ignition point (IP). A stable propane flame (length: 10 cm) was applied at the IP with a torch nozzle-to-sample distance of 2.5 cm. The flame spread process on the exposed surface was recorded using an infrared thermal imager (TiX580, Fluke, Shanghai, China), while the back-surface temperature was monitored using a handheld thermal imager (Ti100, Fluke, China). The test was terminated when the maximum temperature on the back surface reached 275 °C, corresponding to the carbonization temperature of wood fibers [48]. Samples with dimensions of 300 mm × 160 mm × 8 mm were tested. The temperature field on the exposed surface was analyzed using Origin and Smart View 4.4 software. The farthest point from the IP where the temperature reached 300 °C was defined as P_300°C_, and the vertical distance between P_300°C_ and IP was designated as the flame spread distance (D_300°C_). The time required for the back-surface temperature to reach 275 °C (T_275°C_) and the corresponding heating rate were also determined. The charred area on the exposed surface was quantified from images using Photoshop by converting pixel counts into actual area (Sc).

High-temperature charring test: High-temperature charring resistance was evaluated using a muffle furnace (box-type resistance furnace). Groups of six specimens were exposed at 300 °C for 5 min, after which their degree of charring and physical damage were assessed.

Mechanical properties: Mechanical performance was evaluated following GB/T 17657-2022 [49], The flexural strength and modulus of elasticity were measured using the three-point bending method with specimens of 210 mm × 150 mm × 8 mm at a bending test speed of 5 mm/min. The span length between supports was set to 100 mm. Both untreated and post-muffle-treated samples were tested, with six replicates for each condition, and mean values were reported.

Scanning electron microscopy (SEM) and energy-dispersive spectroscopy (EDS): The microstructure and chemical distribution of the modified wood scrimber were examined using a field-emission scanning electron microscope (SU8010, Hitachi, Tokyo, Japan) equipped with an energy-dispersive spectrometer. Prior to observation, samples were oven-dried at 80 °C for 8 h to absolute dryness. Thin sections were cut along both longitudinal and transverse directions, and gold was sputter-coated onto their surfaces. SEM observations were conducted at a resolution of 10 μm with an accelerating voltage of 20 eV to analyze residue morphology, chemical deposits, and char structure.

Fourier-transform infrared spectroscopy (FTIR) analysis: Residual char (20 mg) was ground to a particle size of <2 μm using a mortar mill, then mixed with 200 mg of spectroscopic-grade KBr, homogenized, and dried. The mixture was pressed into transparent pellets at a pressure of 510 × 10^5^ Pa using a hydraulic press. FTIR spectra were collected on a Thermo Scientific Nicolet iS20 FTIR spectrometer (Thermo Fisher Scientific Inc. Waltham, MA, USA) in the range of 500–4000 cm^−1^.

Fire-retardancy and smoke suppression performance: the combustion process of specimens ignited by a constant propane flame was recorded using a digital video camera to provide a direct visual evaluation of their burning behavior and flame-retardant effects. In addition, cone calorimetry was performed in accordance with GB 12441-2018 [50] and ISO 5660:2015 [51] using a cone calorimeter (FTT, Derby UK). Test specimens (100 mm × 100 mm × 5 mm) were wrapped in aluminum foil and exposed to a heat flux of 50 kW/m^2^, with a 25 mm distance from the cone heater. Parameters such as heat release rate and total heat release were recorded, and the fire performance index (FPI) and fire growth index (FGI) were calculated according to Equations (1) and (2) [52].

(1) $$FPI=\frac{TTI}{pk-HRR}$$

(2) $$FGI=\frac{pk-HRR}{T_{pk-HRR}}$$

where TTI is the time to ignition (s), pk-HRR is the peak heat release rate (kW·m^−2^), and T_pk-HRR_ is the time to reach the peak heat release rate (s).

The smoke production behavior of the wood scrimber composites was evaluated using the NBS smoke density test chamber (FTT, UK) according to ISO 5659-2:2017 [53]. Specimens (75 mm × 75 mm × 5 mm) were tested to obtain the specific optical density (Ds) and mass residue as a function of time. The maximum specific optical density (Dsmax) within the given time interval was determined from the Ds–time curve.

X-ray photoelectron spectroscopy (XPS) analysis: The chemical composition and elemental states of the residual char after combustion were analyzed using an X-ray photoelectron spectrometer (ESCALAB 250Xi, Thermo Fisher Scientific, Waltham, MA, USA). Prior to measurement, char residues were ground into fine powder and oven-dried. A monochromatic Al Kα X-ray source (hν = 1486.6 eV) was employed for excitation, and spectra were recorded for quantitative and qualitative analysis of surface chemical states.

## 3. Results and Discussion

### 3.1. Flame-Retardant and Smoke-Suppression Performance

**Figure 2:**
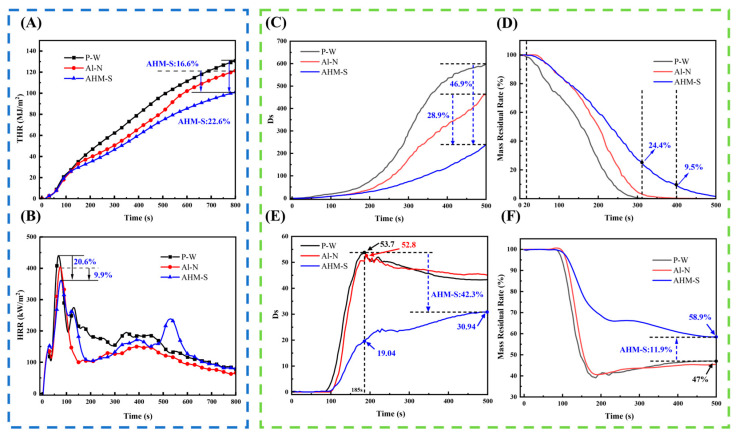
THR (**A**), HRR (**B**) curves of P-W, Al-N, and AHM-S. Specific optical density and mass residual rate curves of P-W, Al-N, and AHM-S under flameless combustion (**C**,**D**). Specific optical density and mass residual rate curves of P-W, Al-N, and AHM-S under flaming combustion (**E**,**F**).

Cone calorimetry was conducted to evaluate the combustion behavior of the composites under a heat flux of 50 kW·m^−2^. The results are shown in Figure 2. The HRR curves (Figure 2B) revealed that all samples exhibited a sharp heat release peak between 80 and 100 s, corresponding to the first peak heat release rate (pk-HRR1), followed by a decline and subsequent increase to a second peak (pk-HRR2). The pk-HRR1 of AHM-S was 403.1 kW·m^−2^, which was 12.47% lower than that of P-W but about 10.2% higher than Al-N. Notably, AHM-S displayed a rapid decline after pk-HRR1 and only gradually increased from 150 to 400 s, whereas P-W and Al-N exhibited a sharp rise to pk-HRR2 at 120 s. After 400 s, AHM-S continued to decline and stabilized at the lowest HRR among the samples, while Al-N exhibited an additional third HRR peak at 530 s. The THR curves (Figure 2A) indicated that at 800 s, the THR of AHM-S was 200.8 MJ·m^−2^, which was reduced by 22.6% and 16.6% compared with P-W and Al-N, respectively. These trends suggest that AHM-S effectively suppressed heat release after the initial combustion stage, delaying the occurrence of pk-HRR2, while P-W and Al-N released heat repeatedly at high intensities, increasing fire risk. The TSR values further demonstrated the smoke suppression capability of AHM-S, which reached 37.6 m^2^·m^−2^, showing reductions of 6.7% compared with P-W and 4.0% compared with Al-N. This confirms that AHM-S provides both flame-retardant and smoke-suppression performance [54]. The detailed cone calorimetry parameters are summarized in Table 2. All samples ignited within 15 s. The FGI values for P-W, Al-N, and AHM-S were 2.12, 2.06, and 0.38, respectively, while the corresponding FPI values were 0.036, 0.042, and 0.094. Compared with Al-N (treated with commercial ammonium sulfate and aluminum hydroxide), the FGI of AHM-S decreased by 81.5%, indicating a much lower fire growth rate. Therefore, AHM-S exhibits higher safety and reliability under realistic fire scenarios, surpassing the performance of widely used flame retardants such as (NH_4_)_2_SO_4_ and Al(OH)_3_.

During the radiative exposure of the material to flame, the outermost structure is initially damaged. Due to the limited thickness of the flame-retardant polyethylene film, the outer veneer and its structure decompose rapidly under high temperature, resulting in a high Heat Release Rate (HRR); nevertheless, this also contributed to distinct flame inhibition at the early stage of combustion. According to scanning electron microscopy observations, the flame-retardant polyethylene film attached to the outer surface of the plywood layer formed a swollen char layer that effectively impeded flame penetration into the interior. Between 100 and 150 s, the HRR curve of AHM-S decreased significantly to its minimum value, and the second peak HRR (pk-HRR2) at 400 s was considerably lower than the first peak (pk-HRR1), further supporting the conclusions described above.

#### NBS Smoke Density Test Analysis

Toxic smoke is a critical factor contributing to casualties in fire accidents. The NBS smoke density test was conducted to simulate real fire conditions, with results summarized in Table 3. The maximum specific optical density (Dsmax) and specific optical density at 500 s (Ds500) were obtained under both flaming and non-flaming conditions.

Under non-flaming conditions, AHM-S exhibited a Dsmax of 329.8, reduced by 28.9% and 46.9% compared with Al-N and P-W, respectively. Under flaming conditions, the Dsmax of AHM-S was 30.97, which was 41.3% and 42.3% lower than Al-N and P-W, respectively. Notably, Al-N showed little improvement compared with P-W, indicating that the addition of Al(OH)_3_ and (NH_4_)_2_SO_4_ did not effectively suppress smoke.

As shown in Figure 2C,D, under non-flaming conditions, the Ds increased steadily within 500 s, with a more pronounced growth rate after 200 s. The mass residue decreased sharply between 20 and 300 s, while AHM-S maintained 9.5% of its initial mass at 400 s, demonstrating superior thermal stability and flame retardancy.

Under flaming conditions (Figure 2E,F), Ds values rose sharply within 80–180 s, corresponding to intense combustion and rapid mass loss. After 180 s, Ds values of Al-N and P-W declined due to char formation, whereas AHM-S continued releasing smoke more slowly, this was attributed to its synergistic flame-retardant system, which suppressed thermal decomposition and formed an intumescent char layer that insulated heat and oxygen, retarded flaming combustion, and shifted burning toward smoldering with gradual smoke release. At 185 s, Dsmax values for P-W and Al-N were more than 60% higher than AHM-S (19.04). At 500 s, the mass residue of AHM-S was 58.9%, 11.9% higher than P-W, confirming its superior char yield and thermal stability.

### 3.2. Flame Propagation and Penetration

**Figure 3:**
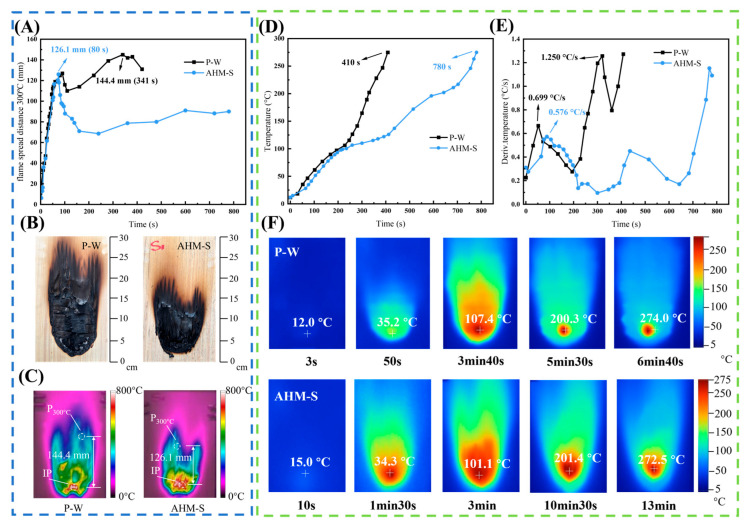
P-W, AHM-S fire spread distance-time curve (**A**), residue images (**B**), and infrared images (**C**). Temperature profiles (**D**), rate-of-rise profiles (**E**), and infrared images (**F**) of P-W and AHM-S backfires.

Under fire conditions, rising temperatures, heat diffusion, and flame spread pose severe safety risks. Thus, examining surface flame spread and longitudinal penetration in flame-retardant wood scrimber/plywood composites is essential. Experiments were conducted on a small-scale flame spread testing platform to evaluate the differences in flame propagation between AHM-S and P-W. The flame spread distance D_300°C_ at different time intervals after ignition on the exposed surface of the materials, as well as the maximum flame spread distance MD_300°C_ and its corresponding time, are presented in Figure 3A. The post-combustion appearance of the specimens is presented in Figure 3B, while the infrared image of the fire-exposed surface at MD_300°C_ is shown in Figure 3C.

The results indicate that after ignition, the D_300°C_ of both P-W and AHM-S increased rapidly during the initial combustion stage, reaching 125 mm and 126.1 mm at 80 s, respectively. However, as the combustion progressed, the D_300°C_ of P-W further increased to a maximum value of 144.4 mm at 380 s. In contrast, the D_300°C_ of AHM-S initially rose rapidly due to the ablation of the flame-retardant coating and the heat radiation absorbed by untreated veneer layers. During the mid-combustion stage, flame penetration was effectively suppressed by the flame-retardant scrimber core and the action of silicon- and boron-based inorganic salts, resulting in stabilization with only minor fluctuations, thus demonstrating a significant flame-retardant effect. Figure 3B shows the residual morphologies, where distinct ash accumulation appeared near the ignition point (IP) for both materials. Notably, the flame-affected area of P-W was considerably larger than that of AHM-S. The actual charred area of AHM-S was 146.8 cm^2^, approximately 37.0% lower than that of P-W (233.2 cm^2^). Figure 3D,E display the back-surface temperature and heating rate evolution of P-W and AHM-S under sustained ignition, along with the time required to reach 275 °C (T_275°C_) and the infrared thermal images of the back surfaces (Figure 3F). The T_275°C_ of P-W was 410 s, whereas that of AHM-S was prolonged to 780 s, representing an improvement of 90.2%, indicating superior resistance to flame penetration. Both materials experienced four stages of back-surface temperature evolution: (i) 0–10 s, negligible temperature change; (ii) 10–100 s, rapid temperature rise accompanied by the highest heating rate; (iii) 100–200 s, heat diffusion across the surface; (iv) after 200 s, continuous longitudinal heat transfer along the thickness. Compared with P-W, AHM-S exhibited a 17.6% lower heating rate in stage (ii) (0.576 °C/s) and a 7.7% reduction at T_275°C_.

Infrared thermal images in Figure 3F further confirm the delayed flame penetration. The back-surface temperature range initially expanded and later contracted. For P-W, the maximum back-surface temperature reached 200 °C (the threshold for wood pyrolysis) at 330 s, while AHM-S required 660 s to reach the same temperature, effectively doubling the delay time and markedly suppressing flame penetration into the interior.

### 3.3. Flame-Retardant Mechanism Analysis

**Figure 4:**
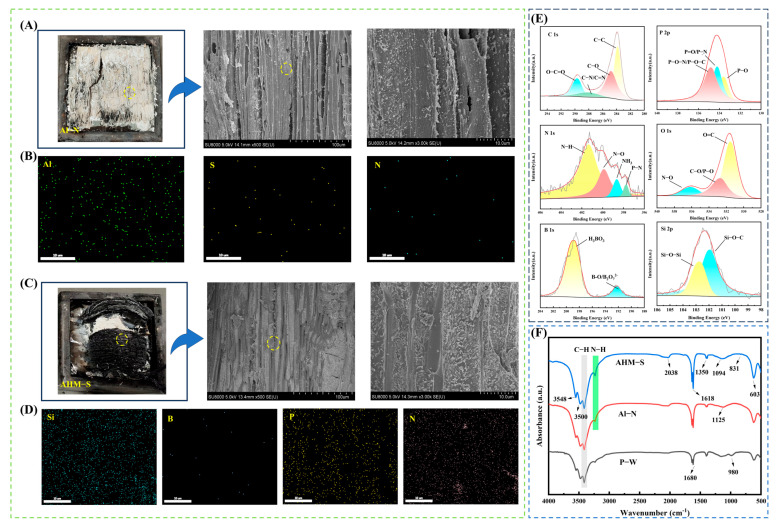
SEM and EDS images of char residue of Al-N after conical calorimetry testing (**A**,**B**). SEM and EDS images of char residue of AHM-S after conical calorimetry testing (**C**,**D**). XPS image of char residue of AHM-S after cone calorimetry test (**E**). FTIR curves of char residue of P-W, Al-N, and AHM-S after conical calorimetry test (**F**).

SEM and EDS analysis: Char residues collected from the interface between the surface plywood and the wood scrimber core after cone calorimetry were analyzed by SEM/EDS (Figure 4). The char from Al-N appeared fibrous and grayish, with no fixed shape, indicating high ash content and almost complete consumption of the wood’s organic components, as the combustion reaction proceeded relatively thoroughly. SEM images showed only a few fragmentary deposits loosely attached to fiber walls and cell lumens, with the wood bundles severely deformed and collapsed at ×500 magnification. EDS spectra confirmed the presence of abundant Al, N, and S, corresponding to residual (NH_4_)_2_SO_4_ and Al(OH)_3_.

In sharp contrast, the char of AHM-S was hard, swollen, and deep black with minimal ash, reflecting an incomplete combustion process in which part of the wood’s organic matter underwent partial oxidation and carbonization rather than full degradation. SEM images revealed dense flake-like deposits tightly adhering to fiber walls and extensively filling cell lumens, forming a compact protective layer. These deposits, composed of phosphorus–nitrogen and silicon–boron compounds, were not only distributed on fiber surfaces but also penetrated into the inner walls of wood bundles, indicating a deep and firm chemical integration with the substrate. At ×3000 magnification, the wood fibers remained aligned and structurally preserved, with EDS spectra detecting abundant P, N, Si, and B, among which Si- and P-rich compounds dominated, confirming the synergistic protection of the flame-retardant system.

FTIR analysis of char: The FTIR spectra of char residues (Figure 4F) revealed enhanced absorption at 3548 cm^−1^ (H_2_O), indicating dehydration and release of water vapor during AHM-S combustion. The -NH_2_ stretching peak (3500 cm^−1^) confirmed the decomposition of melamine, while the band at 3403 cm^−1^ reflected retained wood structure. The strong C=O peak at 2038 cm^−1^ in AHM-S demonstrated formation of new carbonyl groups, contributing to char stabilization. Characteristic bands at 1618 cm^−1^ (C=N), 831 cm^−1^ (P-O-C/C-N), and 603 cm^−1^ (O-N=O) confirmed crosslinking reactions among AHM, MEL, and the wood matrix. Si–N (1094 cm^−1^) and B–N (1350 cm^−1^) bonds indicated chemical interactions between the Wood scrimber core and the modified polyethylene adhesive film, yielding thermally stable structures.

XPS analysis of char: XPS spectra (Figure 4E) confirmed the presence of flame-retardant elements (P, N, Si, B) in the char. In the C 1s spectra, peaks at 283.8 eV (C–O), 284.8 eV (C-C), 288.2 eV (C-N/C=N), and 289.4 eV (O–C=O/P–O–C) indicated crosslinking between melamine/AHM and the wood matrix, introducing nitrogen and enhancing thermal stability [55]. The O 1s spectra revealed peaks at 531.3 eV (C=O) and 536.2 eV (N-O), reflecting reactions between C-N species and wood hydroxyl groups. The Si 2p spectra displayed Si-O-C (102.7 eV) and Si-O (101.8 eV) peaks, confirming formation of polysilicate networks and Si–N bonds during pyrolysis. The P 2p spectra showed P-O (133.4 eV), P-N (134.1 eV), and P-O-N/P-O-C (134.7 eV), indicating formation of stable phosphate structures that promoted char yield [56,57]. The B 1s peak at 192 eV corresponded to B–O and B–N bonds, confirming that boron compounds facilitated dehydration and strengthened the glassy char layer [58]. The N 1s spectra further identified N-H, N-O, N-P, and B-N bonds, with the P-N-C network providing structural reinforcement of the char [59,60,61].

The flame-retardant mechanism of the wood scrimber/plywood composite involves a layered synergistic effect. The outer intumescent system (AHM/MEL/SA) reacts first, producing a swollen char layer enriched with P-N-C linkages that blocks heat and oxygen transfer. Concurrently, inert gases are released and free radicals are trapped, slowing combustion. At deeper layers, sodium silicate melts to form a glassy mineral barrier, while borates decompose to generate boron oxides, enhancing char strength and reducing smoke. The synergy between the surface and core systems is evident from the formation of Si-N and B-N bonds, as confirmed by XPS, which further stabilize the char network. Meanwhile, PO· radicals released by AHM work in concert with steam from silicates and borates to suppress volatile release, thereby improving flame retardancy and smoke suppression.

### 3.4. Application Performance Analysis

**Figure 5:**
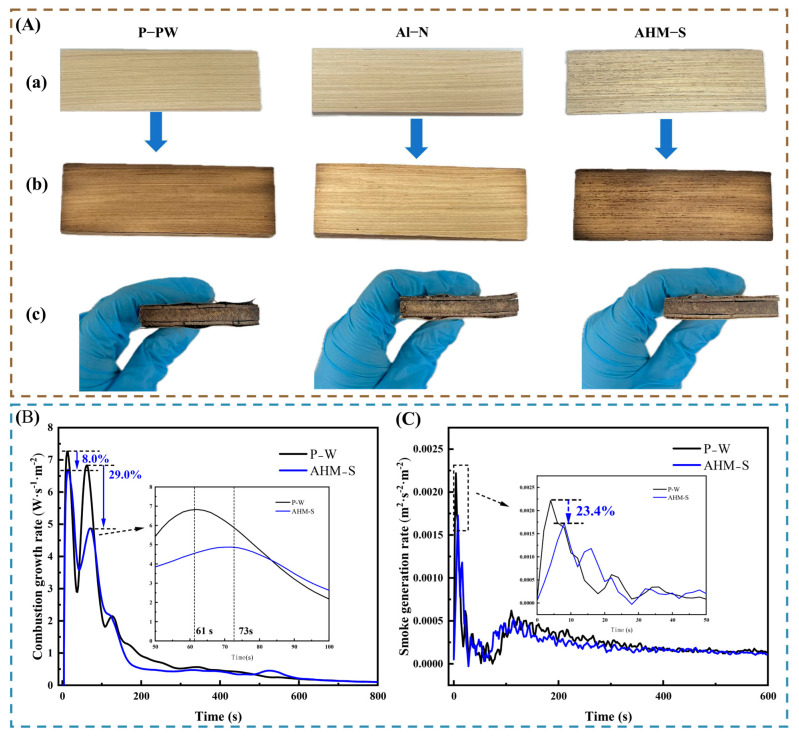
Pure samples and flame-retardant composites before and after treatment in a muffle furnace (**A**). Before performing muffle furnace testing (**a**). After performing muffle furnace testing (**b**). Top view after performing muffle furnace testing (**c**). Combustion growth rate (**B**) and smoke generation rate (**C**) curves of P-W and AHM-S.

The flexural strength tests were conducted to investigate the effect of different flame-retardant formulations on the mechanical performance of the composites. Considering that the loss of structural integrity during fire often leads to severe secondary hazards, the present study further evaluated the mechanical retention of Wood scrimber/plywood composites under elevated temperatures, with a focus on modulus of rupture (MOR) and modulus of elasticity (MOE) before and after muffle furnace treatment. The results are presented in Figure 5B,C, and the key data are summarized in Table 4.

#### 3.4.1. Mechanical Properties at Ambient Temperature

Before thermal treatment, AHM-S exhibited a MOR of 108.51 MPa and MOE of 12.95 GPa, which were 8.5% and 17.9% higher than those of the control sample (100.01 MPa and 10.98 GPa). Compared with Al-N, prepared with commercial flame-retardant formulations, AHM-S showed further enhancements of 35.7% in MOR and 64.6% in MOE, highlighting its superior application potential.

Previous studies have also demonstrated improvements in the mechanical performance of wood composites via different treatments. For example, MMA-hardened hybrid poplar showed mechanical properties comparable to certain hardwood species [62]. Wei et al. [63] optimized the defibration process and obtained composites with MOR = 100 MPa and MOE = 11.3 GPa. Lin et al. [64] produced wood scrimber with MOR = 107 MPa and MOE = 12.7 GPa using high-pressure impregnation. In comparison, the flame-retardant modified composites in this study reached similar performance levels. From the perspective of board configuration, replacing the plywood core with Wood scrimber significantly enhanced the overall strength. Moreover, the mechanical performance of Al-N was lower than that of the control, suggesting that commercial high-performance flame retardants may adversely affect structural strength.

#### 3.4.2. Mechanical Properties After High-Temperature Exposure

After muffle furnace treatment at 280 °C for 5 min, the samples showed apparent surface darkening and shrinkage (Figure 5A). Compared to P-W and Al-N, AHM-S exhibited less color change, indicating shallower thermal degradation and better thermal stability.

Side-view analysis revealed that P-W suffered severe cracks and delamination within its plywood layers; Al-N also displayed partial interfacial separation and warping, reflecting weakened adhesion caused by commercial flame-retardant polyethylene films. In contrast, AHM-S maintained compact structural integrity, with only minor swelling at the interface between the decorative veneer and the upper plywood layer, consistent with the intumescent flame-retardant mechanism.

After thermal treatment, the MOR of all samples decreased to approximately half of their original values, while MOE dropped to about one-fifth. The order of performance was AHM-S > P-W > Al-N. AHM-S retained a MOR of 59.27 MPa and MOE of 2.18 GPa, which were 10.2% and 20.4% higher than P-W, and 37.6% and 36.7% higher than Al-N, respectively, demonstrating superior fire resistance and load-bearing capacity.

Microscopic analysis indicated that the flame-retardant system in AHM-S penetrated deeply into wood bundles and cell walls, forming a dense cross-linked network that enhanced interfacial bonding [65,66]. By contrast, Al-N showed a sparse and uneven microstructure, while the interaction of (NH_4_)_2_SO_4_ with phenolic resin compromised curing, further weakening its mechanical properties.

According to GB/T 9846-2015 [67], the MOR and MOE of AHM-S before high-temperature treatment were 3.38 and 2.35 times higher than those of plywood, respectively, and after treatment, its MOR remained 1.84 times higher [68], confirming its structural advantage for building applications.

#### 3.4.3. Analysis of FIGRA_Cone_ and SMOGRA_Cone_

Fire growth and smoke production are critical parameters for the application of wood-based composites in buildings. The Fire Growth Rate Index (FIGRA_Cone_) and Smoke Growth Rate Index (SMOGRA_Cone_), defined as the maximum ratio of heat release rate to time and smoke release rate to time, respectively, were calculated based on cone calorimeter data following EN13823:2010 [69,70]. The results are shown in Figure 5B,C.

Both P-W and AHM-S exhibited two distinct peaks during combustion, corresponding to surface ignition and subsequent internal burning. Compared with P-W, AHM-S showed an 8% reduction in FIGRA_Cone_, along with a 12 s delay and lower intensity in the second peak, indicating improved flame resistance. Furthermore, the SMOGRA_Cone_ of AHM-S decreased by approximately 23.4% within 0–800 s, effectively suppressing smoke release.

The layered flame-retardant design—consisting of flame-retardant modified wood scrimber in the core and flame-retardant polyethylene film-bonded plywood veneers on the surface—not only balanced load-bearing and decorative functions but also improved fire safety through synergistic effects. This structure promoted the formation of protective char layers, suppressed volatile release, and enhanced resistance against flame penetration. Consequently, AHM-S displayed reduced FIGRA_Cone_ and SMOGRA_Cone_, providing a safer performance profile for building materials. In particular, it holds potential for applications in public buildings, urban complexes, and high-rise structures, where early fire control and structural stability are crucial.

## 4. Conclusions

A wood scrimber/plywood composite fire-retardant material (AHM-S) was developed by combining a silicon–boron modified core with a polyethylene film containing an AHM–melamine–sodium alginate intumescent system in the veneer layers.

AHM-S showed markedly improved fire resistance, smoke suppression, and mechanical performance. Compared with the control (P-W) and commercial formulation (Al-N), pk-HRR2 decreased by 44.1% and 41.5%, while Dsmax dropped by 46.9% and 28.9%. In flame-spread tests, the D_300°C_ was reduced by 18.3 mm and the backside heating rate by 17.6%. The MOR and MOE reached 108.51 MPa and 12.95 GPa, exceeding P-W by 8.5% and 17.9%; after 280 °C exposure, they remained 10.2–20.4% higher than P-W and 36.7–37.6% higher than Al-N.

The superior flame retardancy and smoke suppression properties of the AHM-S sample are primarily attributed to the synergistic effects of its multi-component flame-retardant system. The intumescent system formed by ATMP, HEDP, and MEL generates a compact char layer during combustion, which effectively insulates heat and oxygen while suppressing flaming combustion. Meanwhile, MEL decomposes to release inert gases that dilute combustible gas concentrations, and sodium alginate further enhances the continuity of the char layer through the formation of carbonaceous structures. Additionally, sodium silicate and sodium tetraborate contribute to an inorganic cross-linked network within the wood matrix, improving thermal stability and inhibiting the release of pyrolysis products. Experimental results demonstrate that the AHM-S sample exhibits a reduction in maximum smoke density by over 60%, an increase in residual mass by nearly 12%, a significant decrease in the temperature rise rate on the unexposed surface, and an extension of T_275°C_ by approximately 90.2%. These findings confirm its outstanding performance in inhibiting combustion decomposition, reducing smoke emission, and delaying flame penetration.

The enhanced performance originates from a synergistic mechanism: the intumescent veneer system promotes char formation and radical scavenging, while melamine releases inert gases and strengthens a P–N cross-linked network. In parallel, silicon–boron salts form dense cross-links within the core, improving thermal stability and resistance to flame penetration. AHM-S samples combines high fire safety with strong mechanical integrity, showing promise for use in interior decoration, public facilities, and structural components with high fire-resistance requirements.

## Figures and Tables

**Table 1:** Formulations for layered flame-retardant treatments.

Samples	Surface-Layer Flame Retardant			Core-Layer Flame Retardant	
Pure wood (P-W)	-			-	
Al-NS (Al-N)	Al(OH)_3_			(NH_4_)_2_SO_4_	
AHMSa-SsSt (AHM-S)	AHM	MEL	Sa	Ss	St

**Table 2:** Cone calorimetry data for pure samples and flame-retardant composites.

Sample	T_pk-HRR1_ (s)	pk-HRR_1_ (kW/m^2^)	T_pk-HRR2_ (s)	pk-HRR_2_ (kW/m^2^)	THR (800 s) (MJ/m^2^)	TSR (800 s) (m^2^/m^2^)	TTI (s)	FGI	FPI
P-W	67	460.5	129.1	273.3	130.2	40.3	10	2.12	0.036
Al-N	75	365.8	126.5	260.1	120.9	39.2	11	2.06	0.042
AHM-S	78	403.1	390.4	148.1	100.8	37.6	14	0.38	0.094

Notes: (1) T_pk-HRR1_ represents the time to reach the first peak of heat release rate; (2) pk-HRR1 represents the first peak value of heat release rate; (3) T_pk-HRR2_ represents the time to reach the second peak of heat release rate; (4) pk-HRR2 represents the second peak value of heat release rate; (5) THR denotes the total heat release; (6) TSR denotes the total smoke release; (7) TTI denotes the time to ignition; (8) FPI refers to the fire performance index; (9) FGI refers to the fire growth index.

**Table 3:** Maximum specific optical density and specific optical density at 500 s for P-W, Al-N, and AHM-S.

Samples	Non-Ignition		Ignition	
	Ds_max_	Ds (500 s)	Ds_max_	Ds (500 s)
P-W	621.4	621.4	53.70	43.04
Al-N	464.3	464.3	52.80	45.01
AHM-S	329.8	329.8	30.97	30.97

Notes: (1) Ds_max_ represents the maximum smoke density; (2) Ds (500 s) refers to the specific optical density at 500 s.

**Table 4:** Static flexural strength and modulus of elasticity of pure samples and flame-retardant composites before and after treatment in a muffle furnace.

Samples	MOR (Mpa)	ΔMOR (%)	MOE (Mpa)	ΔMOE (%)
P-W	100.01	46.2	10,982	83.5
P-W(BM)	53.77		1809	
Al-N	79.94	46.1	7866	79.7
Al-N(BM)	43.08		1593	
AHM-S	108.51	45.3	12,946	83.1
AHM-S(BM)	59.27		2178	

Notes: (1) BM indicates the composite material treated in a muffle furnace; (2) ΔMOR represents the reduction rate of static MOR after muffle furnace treatment; (3) ΔMOE represents the reduction rate of MOE after muffle furnace treatment.

## Data Availability

The original contributions presented in the study are included in the article, further inquiries can be directed to the corresponding author.
